# Durability Assessment of Tile-Type Reusable Thermal Protection Materials

**DOI:** 10.3390/ma19020303

**Published:** 2026-01-12

**Authors:** Minjeong Kim, Seong Man Choi

**Affiliations:** Department of Aerospace Engineering, Jeonbuk National University, Jeonju-si 54896, Republic of Korea; gmj1711@jbnu.ac.kr

**Keywords:** reusable thermal protection systems (TPSs), ceramic insulation, borosilicate coating, high-velocity oxygen fuel (HVOF) torch

## Abstract

This study investigates the thermal performance and durability of high-emissivity-coated tile-type insulators, a key material for cost-effective and reusable thermal protection systems (TPSs), using a high-velocity oxygen fuel (HVOF) torch. A single specimen was first exposed to a heat flux of 1.25 MW/m^2^ for 100 s. The specimen exhibited excellent thermal performance, reaching surface temperatures exceeding 900 °C while maintaining an internal temperature increase of less than 20 °C at a depth of 40 mm from the top. Subsequently, a tile-type specimen was subjected to repeated heating at a heat flux of 0.65 MW/m^2^ for up to 100 s. Despite peak surface temperatures exceeding 700 °C, the internal temperature remained stable, within 50 °C, demonstrating consistent thermal protection. Furthermore, under the same heat flux conditions, the results of four repeated experiments confirmed that the temperature of the thermal protection material at a depth of 40 mm from the surface was maintained within a range of ±1.23 °C (95% confidence interval). These results demonstrate that the tile-type TPS maintains reliable thermal protection performance under repeated re-entry conditions.

## 1. Introduction

Reusable spacecraft technology is a core technology that is revolutionizing the economics and efficiency of space exploration and utilization through the recovery and reuse of space launch vehicles. The development of reusable spacecraft has continuously advanced, starting with the development of the X-series aircraft in the 1940s, followed by the X-15 research aircraft in the 1950s, and culminating in NASA’s Space Shuttle, which operated from the 1980s until 2011 [1]. Notably, the Space Shuttle is a prime example, being the first crewed spacecraft to successfully implement the concept of reusability. More recently, reusable technology is being actively researched and commercialized, primarily led by private companies such as SpaceX (with the Falcon 9 rocket and Starship) and the Sierra Nevada Corporation (with the Dream Chaser). Such developments have significantly reduced the cost of space access. They have also expanded the scope of commercial and scientific space exploration.

For the successful execution of a reusable spacecraft mission, the atmospheric re-entry phase is critically important, as it exposes the vehicle to the most extreme environments. When a spacecraft reenters the atmosphere, a hypersonic flow environment with speeds exceeding Mach 10 is generated. The resulting friction between the vehicle and the air produces a high-temperature plasma flow of over 1650 °C around the vehicle, which can critically affect its structural integrity. Therefore, the design and application of a TPS are essential to protect the vehicle from the extreme thermal environment. The aerodynamic heating environment varies depending on the re-entry trajectory, resulting in diverse heat flux distributions and surface temperatures based on the vehicle’s shape. Ensuring reusability under complex thermal conditions, as well as achieving the ultimate goal of reducing launch costs, requires the development of lightweight TPS technologies capable of minimizing system mass and enhancing overall mission efficiency [2].

The materials used in reusable TPS are generally classified into heat-resistant materials and insulation materials based on their function.

The materials used in reusable TPS are generally classified into heat-resistant materials and insulation materials based on their function. Heat-resistant materials, such as C/C composites, C/SiC composites, and ultra-high-temperature ceramics (UHTCs), are typically applied to extreme heat-flux regions, including nose cones, stagnation points, and wing leading edges, where surface temperatures can exceed 1500 °C during atmospheric re-entry. In these regions, the TPS must withstand not only severe loads but also significant aerodynamic and structural stresses. Accordingly, heat-resistant materials are commonly employed in the form of rigid panels, monolithic structural components, or load-bearing external skins, often combined with oxidation-resistant coatings to ensure durability under repeated high-temperature exposure [3,4,5,6,7,8,9,10]. Insulation materials are employed to maintain the structural temperature below an allowable limit (e.g., below 180 °C for the Space Shuttle [11]) in high-temperature environments where the surface temperature exceeds 300 °C. These materials mitigate thermal energy during atmospheric re-entry by re-radiating heat from the surface into the surrounding atmosphere, while simultaneously maintaining low structural temperatures through their inherently low thermal conductivity, which effectively limits heat transfer to the vehicle interior. They are applied as tiles or blankets over large surface areas of the vehicle, such as the windward and leeward sides, which are exposed to relatively lower heat flux conditions. In addition, they serve as secondary supporting layers positioned behind heat-resistant materials, which typically exhibit higher thermal conductivity, to further minimize heat transfer into the airframe. Porous fiber-based insulation materials are most commonly used; representative examples include high-purity silica fibers or ceramic fibers composed of silica and alumina.

Beyond thermal isolation, insulation materials play a crucial role in overall weight reduction, since they cover the largest surface area of the vehicle. Drawing on flight data accumulated over approximately 30 years of Space Shuttle operation, NASA continuously refined its insulation-type TPS, emphasizing performance enhancement and mass reduction. The initial RSI utilized LI (Lockheed Insulation), a low-thermal-conductivity, silica fiber–based material coated with RCG (Reaction Cured Glass). Subsequently, the FRCI (Fibrous Refractory Cured Insulation) material was developed by incorporating alumina-borosilicate fibers, which improved fiber bonding and thereby enhanced mechanical strength [12]. Both insulators employed the RCG coating based on borosilicate glass—renowned for its outstanding heat, chemical, and mechanical resistance—and doped with SiB_4_ as an emissivity-enhancing agent. The RCG coating formed a dense, well-defined boundary between the insulation layer and the surface, but its brittleness made it susceptible to impact damage. To overcome this limitation, the TUFI (Toughened Uni-piece Fibrous Insulation) coating was developed by adding MoSi_2_ to the base composition. The TUFI coating infiltrates into the insulation substrate, resulting in improved surface durability and enhanced impact resistance due to its porous microstructure [13]. Because TUFI coatings were incompatible with existing insulators, the AETB (Alumina-Enhanced Thermal Barrier) material was subsequently developed by adding supplemental alumina fibers to improve durability. However, because AETB exhibited relatively high thermal conductivity, the BRI (Boeing Rigid Insulation) was later designed by removing the alumina-borosilicate fibers altogether [14,15].

The insulation material used on the Space Shuttle’s windward side was manufactured in the form of tiles (RSI, Reusable Surface Insulation) and bonded to the airframe. Owing to the low coefficient of thermal expansion and relative brittleness of RSI, direct attachment to the aluminum airframe could induce failure. Consequently, a substructure such as the SIP (Strain Isolation Pad) was implemented to absorb relative movements between the tile and airframe, thereby minimizing mechanical stresses. The SIP is positioned between the RSI and aluminum structure and is fabricated from needled Nomex aramid fiber, known for its high-temperature stability and superior tensile strength. It is bonded using RTV 560 silicone rubber adhesive, which provides flexibility advantageous for accommodating deformation [16,17,18,19].

A gap of 0.7 mm or greater was maintained between tiles to accommodate thermal expansion differences between the tiles and the airframe, and a Filler bar was positioned underneath this gap. The Filler bar is fabricated as a strip from the same Nomex felt material as the SIP, coated with RTV-560 silicone adhesive, and serves to prevent hot gas intrusion through the tile gap. The Filler bar is attached only to the airframe and not bonded to the RSI, thereby permitting unimpeded thermal expansion [19,20,21].

Reusable TPS can undergo performance degradation due to repeated exposure to high-temperature environments, making durability assessment essential for ensuring reliability over their service life. Previous studies have evaluated the thermal characteristics of TPS and analyzed their behavior under various load conditions. For example, Ajith et al. [22] developed a silica tile-based TPS suitable for application to the RLV-TD (Reusable Launch Vehicle–Technology Demonstrator) and evaluated its performance using the Kinetic Heat Simulation Test Facility at VSSC (Vikram Sarabhai Space Centre) under a maximum heat flux of 4 W/cm^2^. As a result, it was confirmed that, even when the front surface temperature of the tile reached 1300–1400 °C under re-entry conditions, the backwall temperature remained below 120 °C. Milos et al. [23] developed a multi-layer FRSI (Felt Reusable Surface Insulation) system for the leeward region of the Orion Multi-Purpose Crew Vehicle and conducted arc-jet tests and thermal modeling. Using the NASA Johnson Space Center’s arc-jet facility, they simulated ISS (International Space Station) return and lunar return conditions (surface heat flux of 3–40 W/cm^2^) and measured both surface and internal temperatures of specimens consisting of five layers of FRSI bonded to an aluminum substrate with RTV-560 adhesive. Their findings demonstrated that multi-layer felt-type FRSI is experimentally viable as a local ablation-type reusable TPS for re-entry vehicles. Nam et al. [24] manufactured lightweight Al_2_O_3_–SiO_2_ ceramic fiber insulation with a high-emissivity coating and evaluated its high-temperature performance under simulated HVOF conditions. Even after 120 s of exposure to temperatures above 1100 °C, EDS (Energy-Dispersive X-ray Spectroscopy) and XRD (X-ray Diffraction) analyses confirmed excellent microstructural integrity with no specimen failure or deformation, indicating the material’s suitability as a reusable TPS in extreme re-entry environments. Chinnaraj et al. [25] investigated TPS candidate materials for reusable launch vehicles, developing samples comprising Cerakwool substrates with high-emissivity TUFI coatings. These samples were subjected to HVOF torch testing to simulate re-entry conditions at two heat flux levels (0.65 and 1.0 MW/m^2^) for approximately 30 s. All samples maintained interior temperatures well below the TPS backwall design limit (180 °C)—specifically, under 50 °C—and showed no ablation or internal damage upon post-test inspection, thereby demonstrating their strong potential for application as reusable TPS materials.

Reusable thermal protection systems (TPSs) are engineered for repeated use, which requires rigorous evaluation of their long-term reliability and durability. In practical applications, TPS are typically configured as integrated tile assemblies comprising ancillary components such as strain isolation pads (SIPs) and filler bars, making it essential to assess the structural stability of the entire system rather than individual materials alone. In this study, both single block-type specimen and tile-type specimen, representative of real TPS configurations for spacecraft underbody applications, were fabricated using previously verified reusable TPS materials. Repetitive high-temperature environments were simulated using a high-velocity oxygen fuel (HVOF) torch. The resulting physical and chemical changes, along with internal temperature variations under flame exposure, were systematically analyzed to evaluate the thermal protection performance and reusability reliability of the specimens. In contrast to previous studies that primarily focused on the initial assessment of individual TPS materials, the present work provides a comprehensive evaluation of the integrated thermo-structural stability and interfacial reliability of tile-structured TPS under cyclic thermal loading conditions representative of realistic reuse environments. The findings of this study offer critical reliability data for assessing the practical applicability of reusable TPS in operational thermal environments.

## 2. Materials and Methods

### 2.1. Specimens

**Figure 1:**
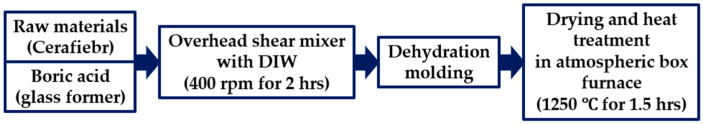
Insulation Material Fabrication Process.

The manufacturing process of the insulator is broadly divided into crushing and mixing, dehydration and forming, drying, and heat treatment. Ceramic fiber (Cerafiber, Morgan Advanced Materials, Windsor, UK [26]), composed of alumina and silica fibers, was used as the starting material. Boric acid, which acts as a binder between the fibers, and distilled water were added, and the mixture was crushed and mixed for 2 h at 400 rpm using an overhead mixer and a metal impeller. The mixture underwent a dehydration and forming process in a cylindrical casting tower and was heat-treated in a box furnace under atmospheric conditions at 1250 °C for 1.5 h, producing cylindrical specimens with a diameter of 60 mm. A simplified diagram of the insulator manufacturing process is presented in Figure 1. All manufactured specimens had the same density of 0.4 g/cm^3^, and the thermal conductivity measured using a Hot Disk TPS 2500S (Hot Disk AB, Goteborg, Sweden) apparatus was 0.075 W/m·K in room temperature.

**Figure 2:**
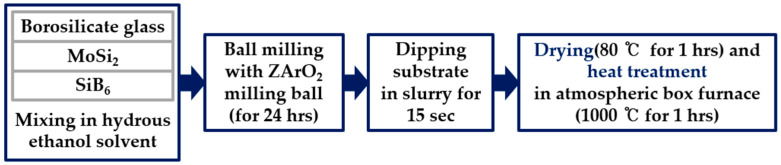
Coating Preparation and Application Process.

The cylindrical insulators were processed according to the specimen design and then coated. The coating applied to the specimens in this study was benchmarked against NASA’s TUFI coating. The borosilicate coating was modified by adding molybdenum disilicide (MoSi_2_) to enhance emissivity. Silicon hexaboride (SiB_6_) was also incorporated to lower the processing temperature and control porosity. The raw materials were prepared as a coating slurry through a 24 h wet ball milling process at 170 rpm with ZrO_2_ balls in an anhydrous ethanol solvent. The prepared coating slurry was applied to the insulator surface using a dip coating method. After coating, the specimens were dried at 80 °C for 1 h and then heat-treated at 1000 °C for 1 h to complete the specimen fabrication. The emissivity was measured using a Fourier Transform Infrared Spectrometer (FT-IR) in the wavelength range of 5–20 μm at a temperature of 50 °C, and the measured emissivity was 0.917. The coating process is presented in Figure 2.

**Figure 3:**
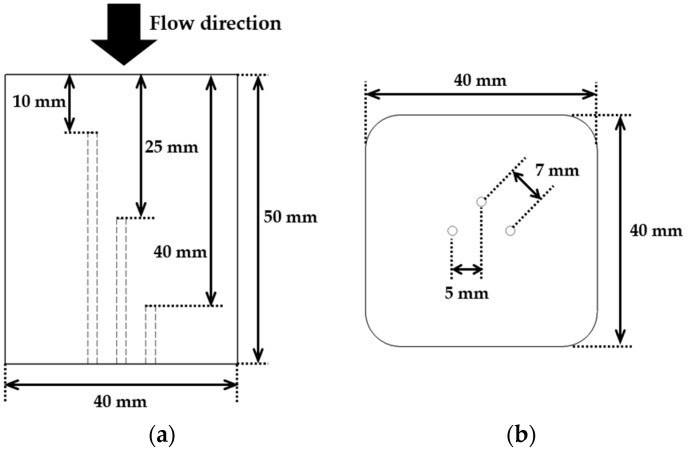
Dimensions of the single specimen: (**a**) side view showing the locations of thermocouples, and (**b**) bottom view indicating thermocouple insertion points.

**Figure 4:**
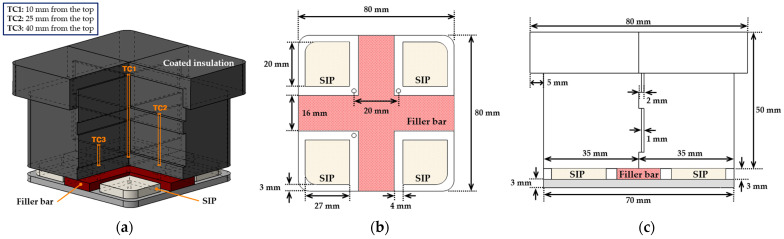
The tile-type specimen configuration and dimensions: (**a**) overall assembly including SIP and filler bar, (**b**) bottom view with gap structure and tile layout, and (**c**) side view showing dimensional specifications.

Figure 3 and Figure 4 show schematic diagrams of the single specimen and the tile-type specimen, respectively.

The single specimen was fabricated as a rectangular prism with dimensions of 40 mm × 40 mm (width × depth) and a height of 50 mm. It was made of a single block of insulating material with rounded edges to facilitate machining and handling, and a high-emissivity coating was applied to its surface. For internal temperature measurement, K-type thermocouples were installed at depths of 10 mm, 25 mm, and 40 mm from the top surface.

For the tile-type specimen, a multi-layered stepped structure was fabricated by assembling four interlocking units. The specimen consisted of three stepped layers, each 2 mm thick, and the interlocking structure between the layers was designed to prevent the penetration of the flame flow into the interior. This configuration was intended to provide mechanically stable joints and to reduce thermal damage under high heat flux conditions. For internal temperature measurement of each specimen, a K-type thermocouple was installed at depths of 10 mm, 25 mm, and 40 mm from the top surface, with each thermocouple spaced 20 mm apart.

To fabricate the tile-type specimen, the SIP and Filler bar were designed and manufactured by simulating NASA’s thermal protection tile attachment structure. These components were bonded to an aluminum plate. The SIP was cut from Nomex needle felt filter cloth (FILMEDIA, Shanghai, China [27]), while the Filler bar was fabricated using the same felt material as a substrate and subsequently coated with RTV-560 silicone (Momentive Performance Materials Inc., Waterford, NY, USA [28]). The RTV-560 silicone was mixed with a curing agent at a ratio of 0.3 wt% to ensure precise proportion control. The RTV-560 mixture was uniformly applied to the felt surface using a brush, followed by a two-step curing process. Room-temperature drying was carried out for 1–2 h under a constant load, and subsequent thermal curing was performed in an electric furnace at 100 °C for 30 min to remove residual solvents and complete the curing reaction.

The fabricated Filler bar and SIP were bonded to the top of the aluminum plate to ensure safety and stable adhesion under high-temperature experimental conditions. The insulating specimen was also attached on top of the SIP. Finally, Cerakwool paper (aluminosilicate-based ceramic fiber insulation, maximum service temperature 1100 °C) was inserted to fill and seal the small gaps caused by surface unevenness at the specimen joints, forming a uniform sealed interface. The material was selected for its low thermal conductivity (≤0.135 W/m·K at 500 °C) and thermal stability under the experimental conditions [29].

### 2.2. Experimental Setup

Testing was conducted using the HVOF system located at the High-Temperature Plasma Application Research Center at Jeonbuk National University. The HVOF system is typically used for thermal spray coating, where powder particles are melted by a high-temperature flame and impacted onto a substrate at supersonic speeds. However, when powder particles are not injected, the system can also be utilized for tests employing a supersonic flame.

**Figure 5:**
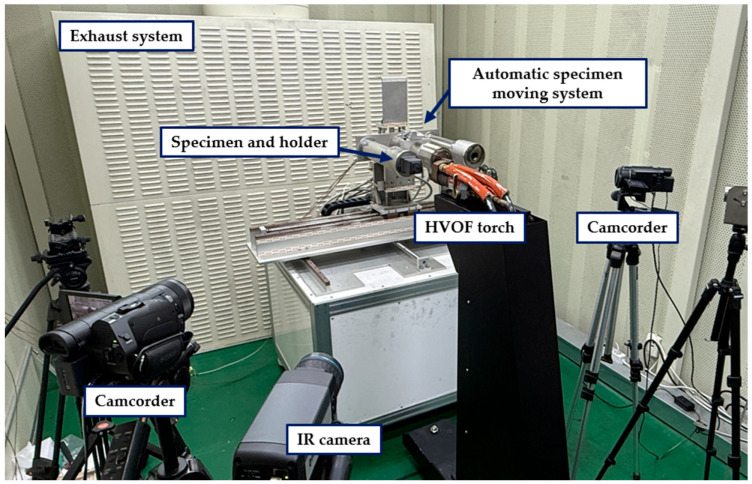
Experimental setup of the HVOF system.

Figure 5 depicts the HVOF system configuration, in which a propane–oxygen mixture is combusted within the chamber to produce a supersonic flame jet that is accelerated through a convergent–divergent nozzle. The maximum flame temperature reaches approximately 2800 °C, and the maximum velocity is around 2100 m/s [30]. During the HVOF tests, each specimen was mounted on a jig integrated with an automated positioning system driven by a motorized XY stage. Prior to each experiment, the nozzle-to-specimen distance corresponding to the target heat flux was determined through preliminary calibration. Based on this calibration, the specimen was initially positioned farther from the nozzle along the x-axis. At the start of each test, the stage was actuated to translate the specimen toward the nozzle at a speed of 150 mm/s, placing it precisely at the predetermined test position. The system motion was controlled using predefined x–y coordinates, ensuring repeatable and accurate specimen alignment for every test.

An infrared (IR) camera was installed around the HVOF gun to measure surface temperatures. Internal temperatures were recorded using K-type thermocouples, and data acquisition was performed with a Compact RIO controller (cRIO-9178) and an NI-9212 input module, both from National Instruments (Austin, TX, USA).

In this study, two types of specimens were tested: the single specimen and the tile-type specimen. The testing conditions were determined based on the heat flux trajectory proposed in a previous study [27], as summarized in Table 1 and Table 2. The Single specimen was evaluated at a heat flux of 1.25 MW/m^2^ for 100 s to assess performance under conditions higher than the nominal environment of 0.58 MW/m^2^. The tile-type specimen was subjected to repeated transient heat loads at a heat flux of 0.65 MW/m^2^, with four exposure durations of 30 s, 30 s, 60 s, and 100 s, to investigate variations in heat transfer characteristics under cyclic thermal loading. Each repeated test was performed following the confirmation that the specimen, exposed to high temperature in the preceding run, had sufficiently cooled down and returned to its initial temperature. Due to the limitations of the HVOF facility, the applied heat flux was fixed at the minimum achievable level of 0.65 MW/m^2^, rather than varied with time to reproduce an actual re-entry heat-flux trajectory.

## 3. Results and Discussion

**Figure 6:**
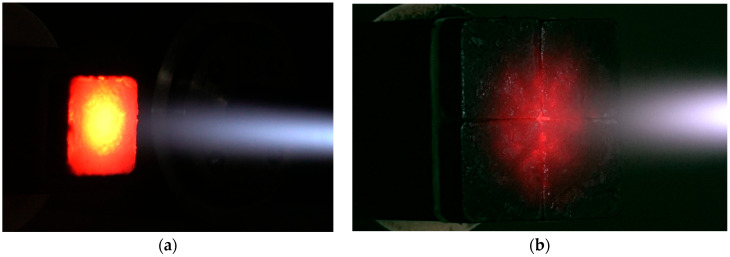
HVOF torch testing images: (**a**) single specimen and (**b**) tile-type specimen.

Figure 6 shows the test setup where the single specimen and the tile-type specimen are exposed to a flame. For the single specimen test, the flame was impinged directly onto the geometric center of the specimen surface. Conversely, in the tile-type specimen test, the flame was applied directly onto the junction between the tiles for evaluation.

**Figure 7:**
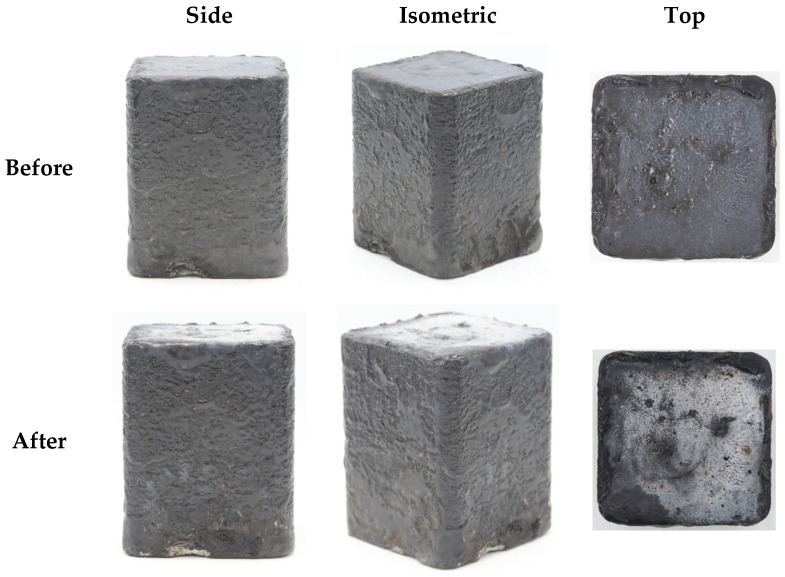
Photographs of the single specimen before and after flame exposure.

Figure 7 compares the macroscopic morphology of the single specimen before and after the test. The results demonstrate that the specimen exhibited superior overall morphological stability, with no significant changes such as ablation or structural failure visually observed. This clearly indicates that the specimen maintained excellent structural integrity under the test conditions. However, a distinct localized color change was observed in the central region of the specimen directly exposed to the flame. This region, initially black before testing, turned white after exposure. This color transition is considered indicative of surface oxidation induced by thermal degradation under prolonged high-heat-flux exposure.

**Figure 8:**
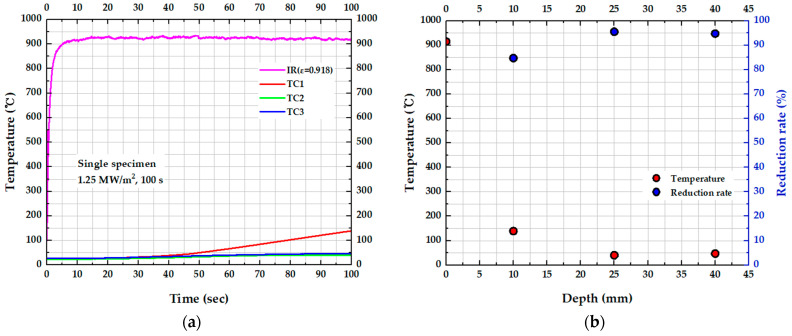
Temperature responses of the single specimen: (**a**) temperature vs. time for surface and embedded thermocouples and (**b**) temperature and reduction rate (relative to surface temperature) vs. depth at test completion.

Figure 8a illustrates the time-dependent temperature variations at the specimen surface (IR) and at three internal positions (TC1, TC2, TC3) over 100 s. During flame exposure, the surface temperature reached a maximum of 934.8 °C and was stably maintained at an average of 915.7 °C. In contrast, the internal temperature of the specimen remained below 150 °C, and minimal temperature variations of only 16.3 °C and 19.5 °C were observed at the internal measurement points TC2 and TC3, respectively, except for TC1, which was located closest to the surface. Figure 8b presents the temperature distribution and the corresponding reduction rate relative to the surface at the end of the test (100 s), as a function of specimen depth. The reduction rate (R(%)) is defined as the percentage of heat prevented from penetrating the insulation, given by

(1) $$R\left(\%\right)=\left(1-\frac{T_{TC}}{T_{surface}}\right)\times 100$$

The reduction rate shows a steep increase near the surface, followed by a gradual rise with increasing depth. Specifically, a sharp temperature drop is observed at TC1 (10 mm), where the internal temperature is reduced by approximately 85% of the surface temperature. At TC2 (25 mm) and TC3 (40 mm), the reduction rate further increased to around 95%. This significant temperature gradient and depth-dependent reduction confirm that the insulation material provides an effective barrier to heat transfer throughout the specimen. However, the internal temperature at TC3 was observed to be approximately 3 °C higher than at TC2. This phenomenon can be attributed to two main factors: (1) direct intrusion of the flame through a small gap between the specimen and the holder, and (2) heat conduction from the heated holder at the bottom surface.

**Figure 9:**
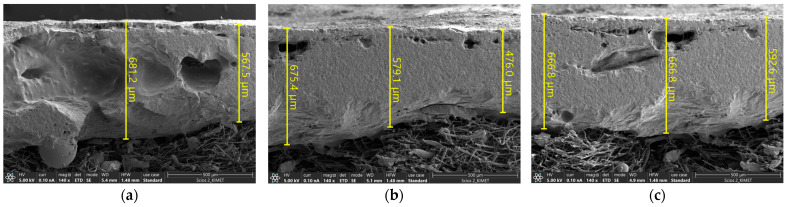
SEM images of coating thickness measured at random points: (**a**) random point 1, (**b**) random point 2, and (**c**) random point 3.

Figure 9 displays FE-SEM (Field Emission Scanning Electron Microscope, Thermo Fisher Scientific, Waltham, MA, USA, Scios 2 HiVac) images of the cross-sectional microstructure after the test. The coating thickness was measured at eight randomly selected points across the coating cross-section, revealing an average thickness of approximately 613 μm. Observation of the cross-sectional microstructure revealed a porous structure. This structural characteristic is similar to that of TUFI coatings, suggesting its potential contribution to improved impact resistance.

**Figure 10:**
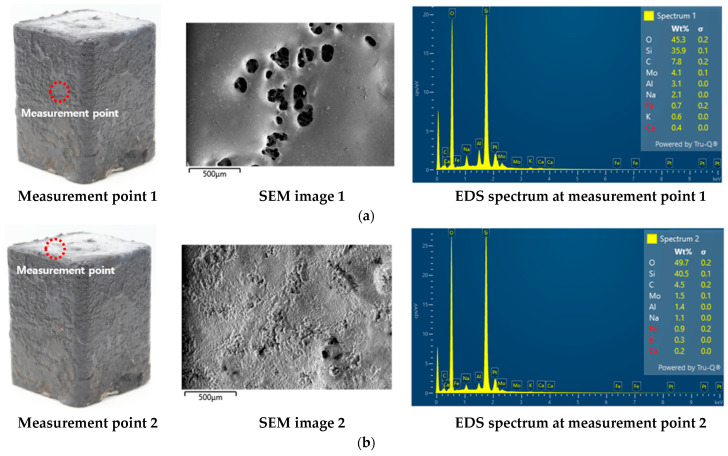
EDS results for (**a**) the coating on the non-exposed surface and (**b**) the coating on the flame-exposed surface.

The EDS (Energy-Dispersive X-ray Spectroscopy, Oxford Instruments, Ultim Max) analysis presented in Figure 10 revealed a difference in the elemental composition between the flame-exposed and unexposed surfaces. The unexposed surface exhibited oxygen (O) and silicon (Si) contents of 45.3 wt% and 35.9 wt%, respectively, whereas the flame-exposed surface contained O 49.7 wt% and Si 40.5 wt%. These results indicate relative increases of 4.4 wt% and 4.6 wt% for O and Si, respectively, on the exposed surface. The simultaneous increase in O and Si on the exposed surface suggests that the Si-containing surface layer underwent oxidation reactions under the high heat-flux flame environment, resulting in the formation of an oxygen-enriched, Si-containing oxidized layer. However, because EDS provides only quantitative elemental information and does not allow identification of crystal structure or phase composition, the specific oxide phase formed on the surface cannot be conclusively determined in this study.

To accurately assess the oxidation level and identify the crystalline structure and phase composition of the formed oxides, follow-up studies employing crystallographic characterization techniques, such as X-ray diffraction (XRD), are required. This approach enables precise identification and quantification of structural changes in the materials.

**Figure 11:**
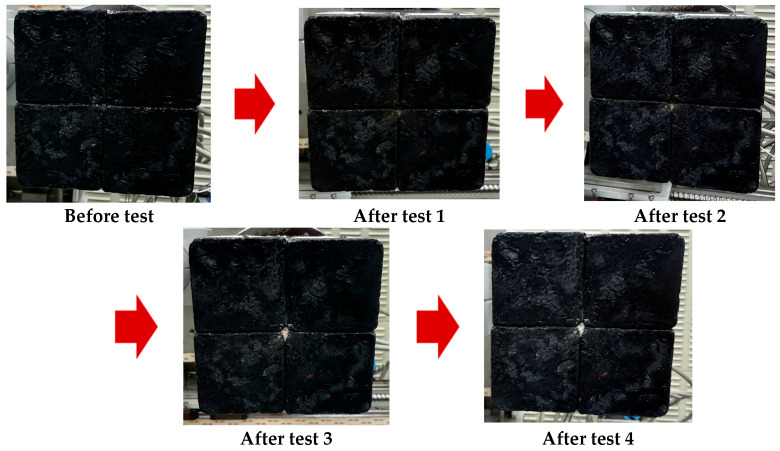
Photographs of the top surface with repeated tests.

**Figure 12:**
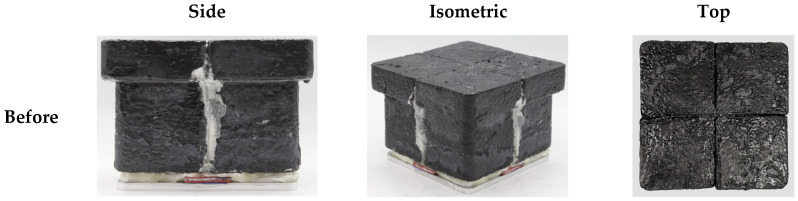

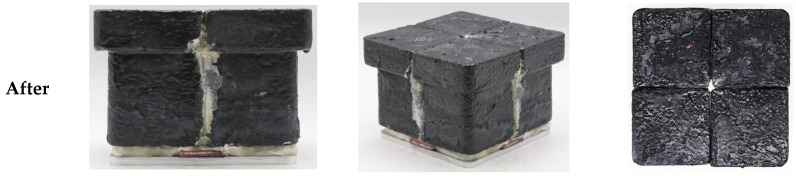
Photographs of the tile-type specimen before and after flame exposure.

Figure 11 shows the surface morphology of the specimen after four repeated test cycles, and Figure 12 compares the overall specimen morphology before and after these repeated tests. Throughout the high-temperature cyclic exposure, no structural physical changes such as separation, abrasion, or damage were observed, and the overall shape remained stable compared to the initial state. Despite this mechanical stability, progressive whitening was clearly observed on parts of the flame-exposed surface, which is indicative of surface oxidation and oxide layer formation due to oxidation reactions at elevated temperatures. Additionally, the ceramic wool paper used to fill the gaps between the tiles was gradually exposed on the surface after repeated testing. These results confirm that repeated high-temperature exposure did not cause the mechanical defects and structural integrity of the specimen but was accompanied by surface oxidation and gradual migration of the filler materials.

**Figure 13:**
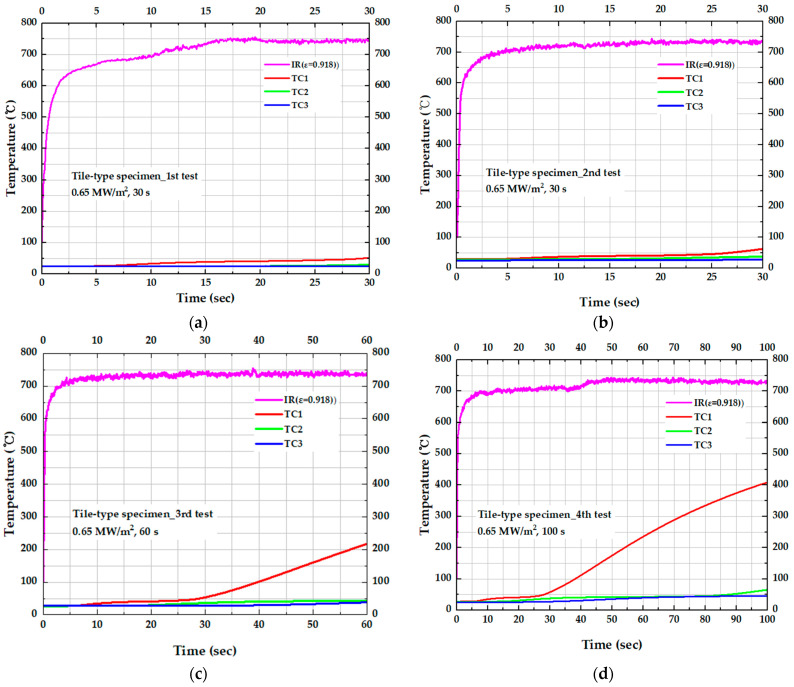
Temperature response for the tile-type specimen in repeated HVOF torch tests: (**a**) 1st test (30 s), (**b**) 2nd test (30 s), (**c**) 3rd test (60 s), and (**d**) 4th test (100 s).

Figure 13 presents the temperature variation in the tile-type specimen over time. Across all tests, the surface temperature followed a similar trend and stabilized at approximately 735 °C. From Figure 13d, the temperatures at TC1 indicates 57.73 °C at 30 s and increased to 408.69 °C at 100 sec. In contrast, the temperatures at TC2 and TC3 in Test 4 remained below 50 °C, comparable to those in the other specimens. These results indicate that, although heat gradually propagates toward the interior with increasing exposure time, the deeper layers remain effectively protected owing to the excellent thermal insulation performance of the tile-type specimen.

Table 3 summarizes the temperature rise ΔT as a function of test duration for each test condition, where ΔT represents the temperature difference before and after the test. Under the most severe test condition, Test 4 (100 s), ΔT at TC1 (10 mm) reached 381.02 °C, indicating that the high surface temperature of 716 °C was transmitted to a significant extent to a depth of 10 mm. In contrast, ΔT at TC3 (40 mm) remained below 22 °C for all test conditions, demonstrating that heat transfer through the insulation layer attenuates sharply with increasing depth. Specifically, under the 30 s test conditions (Test 1 and 2), ΔT at 10 mm was 26.74 °C, whereas at 40 mm it was only 0.24 °C. This reduction with depth highlights the exceptionally high thermal resistance of the insulation structure.

**Figure 14:**
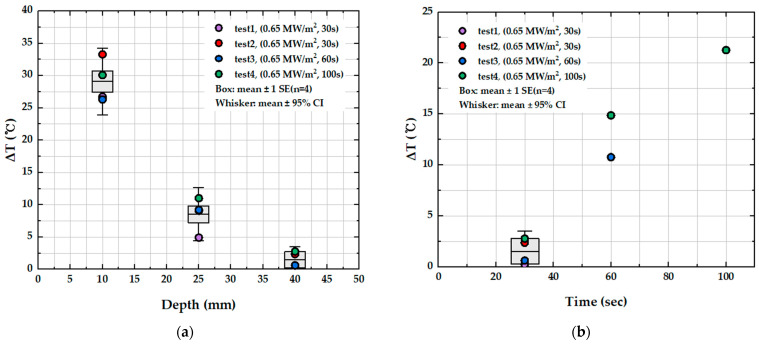
Box-and-whisker representation of temperature rise (ΔT) for the tile-type specimen: (**a**) dependence on thermocouple depth (TC1, TC2, TC3) at the 30 s test duration and (**b**) dependence on test duration. The boxes represent the mean ± 1 standard error, and the whiskers indicate the 95% confidence intervals based on four repeated tests (*n* = 4).

Table 4 compiles the statistical quantities for ΔT at each depth at 30 s obtained from repeated tests (*n* = 4), with these data visualized in Figure 14a. The mean ΔT decreased significantly with depth, from 29.10 °C at 10 mm to 8.56 °C at 25 mm and 1.50 °C at 40 mm. At all locations, the standard deviation was less than 3.26 °C and the standard error was less than 1.63 °C, while the 95% confidence intervals of the mean were also narrow, within ±3.19 °C at TC1 and ±1.23 °C at TC3. These results indicate that the internal temperature response was measured with high repeatability under identical test conditions. The mean ΔT and its uncertainty (±1 standard error and 95% confidence interval) shown in Figure 14a confirm that heat transfer decreases steeply with distance from the heated surface, which is consistent with the expected diffusion-controlled thermal response in the insulation layer as depth increases. Figure 14b further shows that even when the test time is extended to 100 s, ΔT at 40 mm (TC3) remains below 25 °C, indicating that thermal penetration over time is strongly restricted in the deeper region. In other words, while the rate of increase in ΔT at TC1 accelerates nonlinearly as the test time increases from 30 to 60 to 100 s, the effect of test time on ΔT at TC3 is minimal, reflecting pronounced thermal lag at greater depth.

Collectively, these results indicate that the tile-type thermal protection system can limit the temperature rise at a depth of 40 mm to below 22 °C even when the surface temperature exceeds 700 °C, while maintaining high repeatability under identical test conditions. This demonstrates that, despite the inherent segmented architecture, the tiled con-figuration provides sufficient thermal shielding performance and reliability within the specified test envelope. However, due to the intrinsic nature of tiled insulation, small gaps between adjacent tiles are unavoidable. These gaps can act as additional heat-transfer pathways that locally increase both convective and radiative heat fluxes. Therefore, future studies should establish a comprehensive material property database, incorporating accurate high-temperature thermophysical and thermal expansion data, and employ this database as the basis for optimizing gap thickness and tile layout design. Furthermore, quantitative evaluation of the thermal effects and convective heating associated with gap structures under representative reentry environments should be carried out to propose reproducible TPS tile configurations that preserve both structural and thermal integrity, thereby further enhancing the durability and reliability of reusable spacecraft TPS.

## 4. Conclusions

This study experimentally verified the thermal durability of both single and tile-type ceramic TPS specimens under simulated re-entry conditions using an HVOF torch. All specimens maintained their geometric integrity during the tests, confirming their structural stability.

The single specimen (1.25 MW/m^2^, ~100 s) exhibited an internal temperature variation of only 19.5 °C at the 40 mm from the top, even when the surface temperature exceeded 900 °C, demonstrating a strong attenuation of heat transfer through the thickness.

For the tile-type thermal protection system, the temperature rise at 40 mm from the top remained below 25 °C even when the surface temperature exceeded 700 °C, demonstrating significant reduction in heat transfer through the insulation thickness. After 30 s of heating, the mean temperature rise significantly decreased from 29.10 °C at 10 mm to 8.56 °C at 25 mm and 1.50 °C at 40 mm from the top. Furthermore, the 95% confidence intervals constrained within ±3.19 °C near the 10 mm from the top and ±1.23 °C at the 40 mm from the top location, confirming that the internal thermal response was measured with high repeatability and minimal data scatter under identical test conditions.

In conclusion, the present study experimentally verified the outstanding thermal protection capability and structural stability of a reusable ceramic-based TPS, without any significant performance degradation under repeated thermal loading, thereby confirming its durability and reliability. Building on these findings, future work should focus on refined gap-management strategies and the establishment of an enhanced high-temperature material property database, so as to enable optimized TPS lifetime design and guide the development of next-generation reusable spacecraft thermal protection systems.

## Figures and Tables

**Table 1:** Single specimen test conditions.

No.	Specimen Name	Heat Flux (MW/m^2^)	Distance from the HVOF Nozzle Exit (mm)	Duration (s)
1	Single specimen	1.25	200	100

**Table 2:** Tile-type specimen test conditions.

No.	Specimen Name	Heat Flux (MW/m^2^)	Distance from the HVOF Nozzle Exit (mm)	Duration (s)
1	Tile-type specimen	0.65	260	30
2			260	30
3			260	60
4			260	100

**Table 3:** Temperature rise (ΔT) measured at thermocouples (TC1–TC3) during repeated tests on a tile-type specimen under different test durations.

Test Case	Duration (s)	Maximum Surface Temperature (°C)	ΔT (°C)		
			TC1 (10 mm)	TC2 (25 mm)	TC3 (40 mm)
Test 1	30	754.94	26.74	4.93	0.24
Test 2	30	743.31	33.28	9.08	2.37
Test 3	30	753.89	26.30	9.21	0.63
	60		190.38	16.04	10.76
Test 4	30	743.77	30.07	11.02	2.78
	60		207.26	15.74	14.87
	100		381.02	38.53	21.27

**Table 4:** Statistical summary of temperature rise (ΔT) at each thermocouple depth at the 30 s test duration: mean values, standard deviations, and 95% confidence intervals based on four repeated tests.

Thermocouple Location	Depth (mm)	Sample Size, n	Mean ΔT (°C)	Standard Deviation (°C)	Standard Error (°C)	95% Confidence Interval (°C)
TC1	10	4	29.10	3.26	1.63	3.19
TC2	25	4	8.56	2.57	1.29	2.53
TC3	40	4	1.50	1.26	0.63	1.23

## Data Availability

The original contributions presented in the study are included in the article. Further inquiries can be directed to the corresponding author.

## Abbreviations

AETB	Alumina-Enhanced Thermal Barrier
BRI	Boeing Rigid Insulation
C/C	Carbon/carbon
C/SiC	Carbon fiber-reinforced silicon carbide
C/SiOC	carbon-containing silicon oxycarbide nanocomposites
EDS	Energy-Dispersive X-ray Spectroscopy
FE-SEM	Field Emission Scanning Electron Microscope
FRCI	Fibrous Refractory Cured Insulation
FRSI	Felt Reusable Surface Insulation
HETC	High-Emittance Thermal Control
HVOF	High-Velocity Oxygen Fuel
ISS	International Space Station
IXV	Europe’s Intermediate eXperimental Vehicle
LI	Lockheed Insulation
RCC	reinforced carbon–carbon
RCG	Reaction Cured Glass
RSI	Reusable Surface Insulation
RTV-TD	Reusable Launch Vehicle–Technology Demonstrator
SiC	Silicon Carbide
SIP	Strain Isolation Pad
TC	Thermocouple
TPS	Thermal Protection System
TUFI	Toughened Uni-piece Fibrous Insulation
TUFROC	Toughened Unipiece Fibrous Refractory Oxidation-Controlled
UHTC	Ultra-high temperature ceramics
VSSC	Vikram Sarabhai Space Centre
XRD	X-ray Diffraction
